# When size matters: the first comprehensive anatomical study of a species of “Condylocardiidae”, an extremely miniaturized bivalve

**DOI:** 10.7717/peerj.12108

**Published:** 2021-08-30

**Authors:** Flávio Dias Passos, Alan Rodrigo Batistão, Rüdiger Bieler

**Affiliations:** 1 Department of Animal Biology, Institute of Biology, University of Campinas, Campinas, São Paulo, Brazil; 2 Negaunee Integrative Research Center, Field Museum of Natural History, Chicago, IL, United States

**Keywords:** Dwarfism, Small body size, Paedomorphosis, Neoteny, Mollusca, Bivalvia, Histology, Western Atlantic, Archiheterodonta, Carditidae

## Abstract

‘Miniaturization’ is a widespread phenomenon among the Metazoa. In the molluscan class Bivalvia, records of miniaturization are numerous. Among the Archiheterodonta, *Warrana besnardi* (Klappenbach, 1963) has attracted attention for its tiny size, which does not exceed 1.5 mm in shell length, and because it belongs to a group with limited anatomical information and often-debated status, the “Condylocardiidae” (which recent molecular studies place deeply nested within the family Carditidae). All species of *Warrana* Laseron, 1953 are small-bodied, and so miniaturization presumably occurred from a large-bodied ancestor within the Carditidae **sensu* lato*. South American *W. besnardi* is here studied in detail. Its small size and the enlargement of the anterodorsal region during growth, reflects (and likely led) to infaunal habit, living as a burrowing bivalve that passively feeds on deposit particles entering the pallial cavity anteriorly. Mantle glands, previously reported as a common feature of other archiheterodonts, are missing in *W. besnardi*, but spongiform tissue in the antero-ventral portion of the mantle lobes presumably represents a blood sinus that might compensate for the great reduction of the ctenidia. Lecithotrophy is reported, with yolky oocytes bearing a thick non-cellular capsule layer; brooding was not observed, and it is here hypothesized that the extreme miniaturization, with the great reduction of ctenidia, is responsible for a shift in the reproductive mode of condylocardiids, contrasting with the commonly reported ovoviviparity of the carditids.

## Introduction

Miniaturization, the evolution of extremely small adult body size, has been described as a very widespread phenomenon in animals, spanning numerous phyla, with important consequences for both the organismal biology and phylogenetic diversification (*e.g*., Hanken & Wake, 1993). In some cases, such as in the evolution of meiofauna, miniaturization has been described as the rule rather than the exception (Rundell & Leander, 2010). The reduction in body size is often associated with reduction or simplification of anatomical components, and in extreme cases the loss of entire organ systems (Gould, 1977; Higgins & Thiel, 1988). The simplified adult morphology often resembles that of juveniles (or embryos/larvae) of larger-bodied relatives. Such miniaturization has frequently been interpreted as precocious truncation of the ancestral developmental program, resulting in the retardation in somatic development and leading to the retention of juvenile features in the sexually mature adult form (neoteny or progenesis; with the latter lowering age at first reproduction, *e.g*., Gould, 1977). Examples in the Mollusca include shell dwarfism in the land snail genus *Cerion* Röding, 1798 (Gould, 1984) and the retention of byssus glands into adulthood in the small-bodied veneroidean bivalve species *Turtonia minuta* (Fabricius, 1780) (Ockelmann, 1964). As previously discussed by Hanken & Wake (1993) and Rundell & Leander (2010), phenotypic miniaturization combines both ancestral and derived traits, and the anatomical reduction and simplification might be paired with novel structural configurations. Among the life history correlates of miniaturization are a reduction in fecundity and an increase in egg size (*e.g*., Shine & Greer, 1991). Miniaturization apparently addressed a range of different selective pressures in Mollusca. In some cases, *e.g*., in the marine bivalve *Philobrya munita* (Finlay, 1930), the small body size allowed colonization of niches (such as living in crevices or among algal holdfast on rocky shores) outside the ancestral infaunal mode of life (Morton, 1978). In the otherwise surface dwelling Solenogastres, it allowed the colonization of interstitial spaces (*e.g*., García-Álvarez, Urgorri & Cristobo, 2000). Whereas small size facilitates adult dispersal (*e.g*., in *Gemma gemma* Totten, 1834, a venerid bivalve of the western Atlantic that successfully invaded the Eastern Pacific; see Carlton, 1979; Hoos et al., 2010), brooding enhances the likelihood of a successful founder event as has been shown in marine gastropods (*e.g*., Johannesson, 1988; Bieler et al., 2017).

The bivalve clade Archiheterodonta comprises about 415 living species of marine bivalves, and thus is much less diverse than its sister group Euheterodonta with more than 5,000 species (Huber, 2010). Traditionally, archiheterodonts are classified in four families, Crassatellidae Férussac, 1822, Carditidae Férussac, 1822, Astartidae d’Orbigny, 1844, and Condylocardiidae F. Bernard, 1896, whose relationships have long been debated (*e.g*., Boyd & Newell, 1998; Yonge, 1969). Usually, Crassatellidae and Astartidae are placed together in the superfamily Crassatelloidea Férussac, 1822, whereas Carditidae and Condylocardiidae are grouped in the Carditoidea Férussac, 1822 (*e.g*., Chavan, 1969; Slack-Smith, 1998; Bieler, Carter & Coan, 2010; González & Giribet, 2015; Combosch et al., 2017; Lemer, Bieler & Giribet, 2019), with alternative arrangements combining all of them into a single group proposed by other authors (*e.g*., Yonge, 1969; Healy, 1995; Taylor, Glover & Williams, 2005; Carter et al., 2011). Close affinity among Astartidae, Carditidae, and Crassatellidae has been corroborated through comparative studies on their general morphology (Yonge, 1969), and on some particular characters, such as hemoglobin (Terwilliger & Terwilliger, 1985) and sperm structure (Healy, 1995), although species of Condylocardiidae were not included in these latter studies. Through molecular studies, a close relationship of Astartidae and Carditidae was demonstrated by Giribet & Wheeler (2002) and Giribet & Distel (2003), and then the monophyly of Archiheterodonta was supported by the inclusion of the Crassatellidae (Taylor, Glover & Williams, 2005), with the position of Condylocardiidae remaining unknown. The subsequent addition of members of Condylocardiidae corroborated the traditional view that extant archiheterodonts consist of two main clades, Crassatelloidea and Carditoidea (Bieler et al., 2014; González & Giribet, 2015; Combosch et al., 2017; Lemer, Bieler & Giribet, 2019). Relationships among condylocardiids and other archiheterodonts have been discussed since the description of the genus *Condylocardia* by Bernard (1896a, 1896b) who suggested that condylocardiids are related to the Carditidae (Bernard, 1896b), and that their divergence was probably due to paedomorphosis. Debates have also focused on the subdivision of Condylocardiidae, which was divided into two subfamilies, Condylocardiinae F. Bernard, 1896 and Cuninae Chavan, 1969, by Chavan (1969), to which Kuroda, Habe & Oyama (1971) added Carditellinae Kuroda, Habe & Oyama, 1971. However, recent multilocus molecular studies (González & Giribet, 2015; Combosch et al., 2017) found members of nominal condylocardiines (*Carditopsis* E. A. Smith, 1881) and Carditellinae (*Carditella* E. A. Smith, 1881) nested within the family Carditidae, questioning the validity of these groupings. Molecular data for additional nominal condylocardiid genera are needed and, in this study, “condylocardiids” is used informally, as the carditoidean group that includes Condylocardiinae, Cuninae, and Carditellinae.

In the Archiheterodonta, condylocardiids stands out as its most species-rich group, composed of about 150 extant species in 21 genera (Huber, 2010). The group generally differs from carditids **sensu* stricto* by by their minute, suborbicular to trigonal shells, which rarely exceed three mm in length and bear cap-like prodissoconchs. Despite this diversity, investigations on the biology and anatomy of condylocardiids are rare (*e.g*., Mikkelsen & Bieler, 2007; Bieler, Mikkelsen & Giribet, 2013), with only a few notes on the reproduction of some species (see below). Moore (1961) appears to be the only one to have described a living condylocardiid, when he reported specimens of *Goniocuna dalli* (Vanatta, 1904) as occurring in fairly coarse sands off the Mississippi coast, where it lives as an active infaunal bivalve that “ploughs around in the sand several millimeters beneath the surface”. He also noted that one specimen “gave birth to a single young […] which was approximately 300 microns in length (and) immediately began to burrow”, thus suggesting brooding up to the juvenile stage. Brooding was also recorded for a few other species from different genera, and some data on the soft parts were provided along conchological observations in faunistic or taxonomic studies (Bernard, 1896a; Bernard, 1896b, Bernard, 1898; Waller, 1973; Salas & Rolán, 1990; Salas & von Cosel, 1991; Middelfart, 2002a; Middelfart, 2002b; Oliver & Holmes, 2004; Güller & Zelaya, 2013; Malchus & Sartori, 2013).

To address the limited anatomical knowledge of condylocardiids, focus herein is placed on a species that was recently explored for its unusual shell features, *Warrana besnardi* (Klappenbach, 1963) (Batistão & Passos, 2020). This species reaches up to 1.5 mm in shell length, and is the single member of the genus *Warrana* Laseron, 1953 in the Atlantic. The genus encompasses 14 other species in the Pacific Ocean, all of them being extremely small-bodied (less than three mm in shell length; Middelfart, 2002b). Based on the larval shell of *Warrana besnardi*, Batistão & Passos (2020) hypothesized a lecithotrophic development, involving large eggs and brooding.

As pointed out by Hanken & Wake (1993), phylogenetic context should be addressed for the analysis of occurrence of miniaturization in a given lineage. In the case of Archiheterodonta, for which phylogenetic information remains sparse, this is a difficult task. Members of the nominal subfamily Cuninae (where *Warrana* is classified), for example, were missing in the molecular analysis of González & Giribet (2015) and Combosch et al. (2017), and no condylocardiid was included among the taxa sampled by Lemer, Bieler & Giribet (2019). Pérez (2019) performed a shell-morphology-based phylogenetic study of many taxa of Carditidae, including fossils, but condylocardiids, as members of a different nominal family, were not considered. Regardless of the exact position and status of Condylocardiidae or Cuninae, miniaturization has occurred in a lineage that gave rise to the genus *Warrana*. We are exploring the unusual morphological characters observed in *W. besnardi* and discuss whether its miniaturization might have led to a mode of life different from its larger-bodied relatives.

## Materials & Methods

Individuals of *W*. *besnardi* were obtained from the collections of the Museum of Zoology of the University of Campinas (ZUEC). The material originated from bottom samples collected by Van Veen grabs during the activities of the ‘Habitats Project—Campos Basin Environmental Heterogeneity’, conducted in February and July 2009. Sampling was undertaken on the shelf and continental slope of an area of oil and natural gas exploitation of the States of Rio de Janeiro and Espírito Santo, off the southeastern Brazilian coast. During that survey, numerous bottom samples were taken from depths of 12 to 3,200 m and sieved through a 0.5-mm mesh. From 28 of these samples, collected at depths of 16 to 56 m, 102 specimens of *W*. *besnardi* with soft parts were obtained for our study. These had been initially fixed in 4% formalin and then preserved in 70% alcohol.

Using fine needles, each one was dissected, with one of the valves extracted together with its respective mantle lobe. In this way the organs of the mantle cavity were observable in their form and concerning the presence of broods. Aided by the transparency of the tissues, the visceral mass was examined for mature gonads; some oocytes were then extracted and measured. Images were obtained by a camera coupled to a Zeiss SteREO Discovery V8 stereomicroscope.

The structure of the organs of the mantle cavity and the visceral mass were also observed by histological sections. For this purpose five individuals (two females and three males) were decalcified in a solution of 100 ml distilled water containing 0.88 g of NaCl and 1.02 g of ascorbic acid, dehydrated in an ascending series of ethanol, and embedded in methyl methacrylate (Historesin^®^); through serial microtomization. The obtained five µm thick histological sections were stained in toluidine blue. All the males and one female were sectioned in transverse planes; the other female was sectioned in parasagittal/sagittal planes. All of the sectioned specimens measured ca 1.1 mm in shell length. Sections were photographed with a camera coupled to a Leica DM4 B microscope.

Because of the sampling protocol used during the survey project, which involved formalin fixation, molecular sequencing techniques could not be employed.

## Results

**Figure 1:**
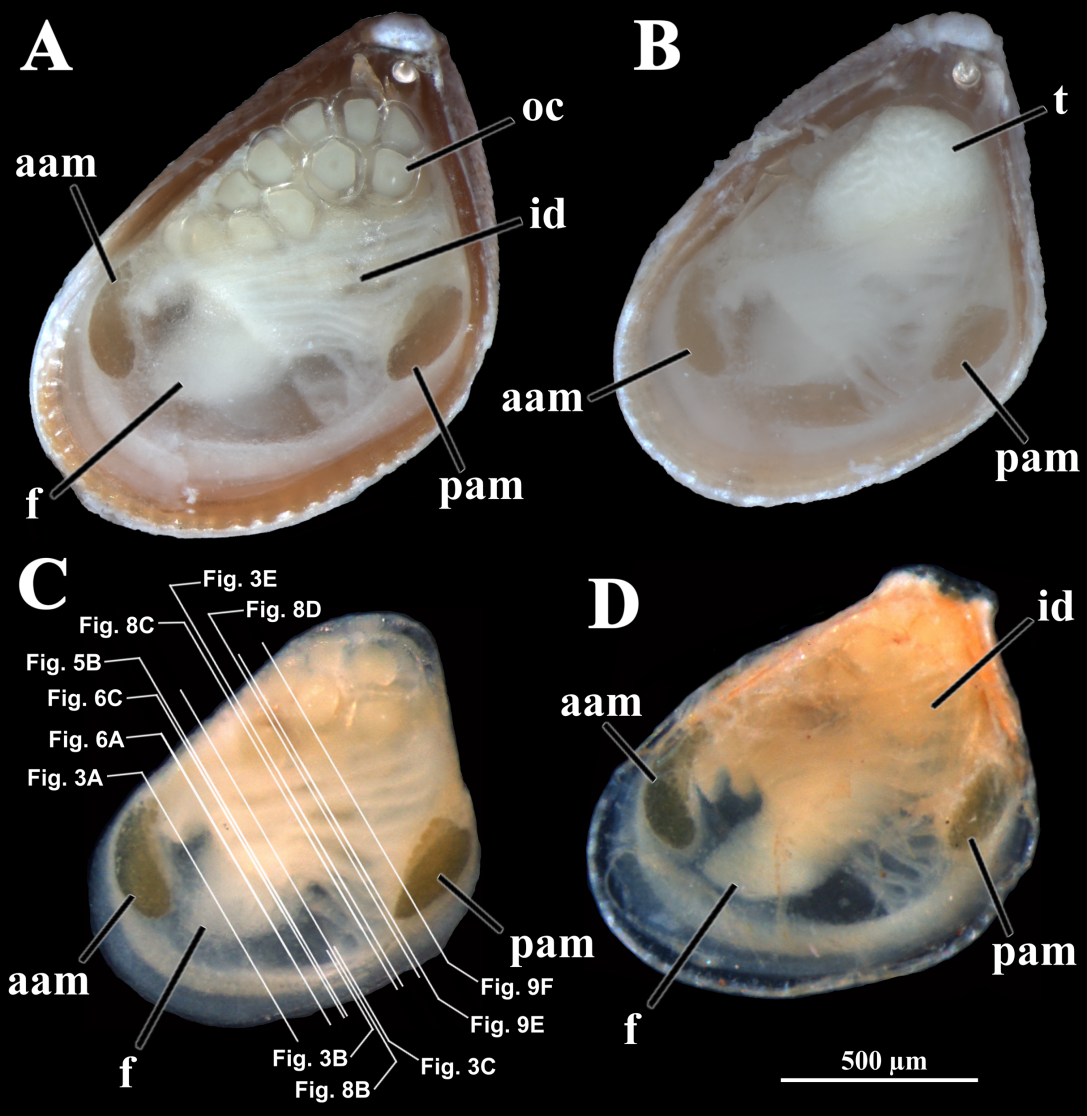
*Warrana besnardi*—Female (A, C, D) and male specimens (B), as viewed from the left side. A and B with the left valve removed by dissection (left mantle lobes still present), C and D with both valves decalcified and the animals used for histological sections (C sectioned through transversal plane; D through parasagittal and sagittal planes). Planes indicated in C correspond to histological sections in Figs. 3A–3C, 3E, 5B, 6A, 6C, 8C, and 9E. Sections of Figs. 8B, 8D and 9F stem from a male and here the section planes are projected on the specimen in 1C. Abbreviations: aam, anterior adductor muscle; f, foot; id, inner demibranch; oc, oocyte inside the ovary; pam, posterior adductor muscle; t, testis.

**Figure 2:**
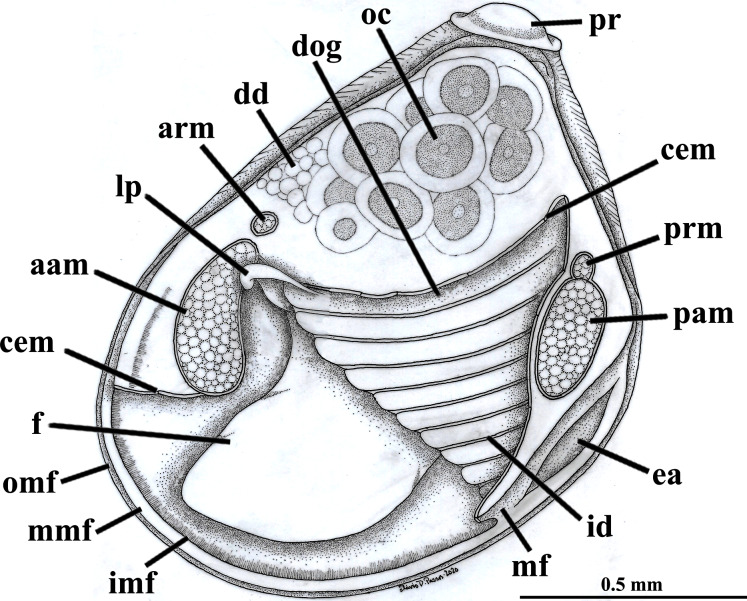
*Warrana besnardi*—Anatomy, with focus on the pallial cavity, as viewed from the left side after removal of the left shell valve and mantle lobe. Abbreviations: aam, anterior adductor muscle; arm, anterior retractor muscle; cem, cut edge of mantle; dd, digestive diverticulum; dog, distal oral groove; ea, exhalant aperture; f, foot; id, inner demibranch; imf, inner mantle fold; lp, labial palp; mf, mantle fusion; mmf, middle mantle fold; oc, oocyte inside the ovary; omf, outer mantle fold; pam, posterior adductor muscle; pr, prodissoconch; prm, posterior retractor muscle.

Four of the dissected and microtomized specimens of *Warrana besnardi* are shown in Figs. 1A–1D. Based on the observation of these and all other individuals, the mantle cavity with its organs as viewed from the left side is illustrated in Fig. 2. Due to the antero-ventral elongation of the shell that occurs with growth (as pointed out by Batistão & Passos, 2020), the umbones are remarkably shifted posteriorly, resulting in the strong inequilateral shell form. Despite this strong inequilaterality, the adductor muscles are quite similar, both being large and elongate but subequal in size and shape, the anterior one reniform in cross section and slightly larger than the oval posterior one (Figs. 1 and 2).

**Figure 3:**
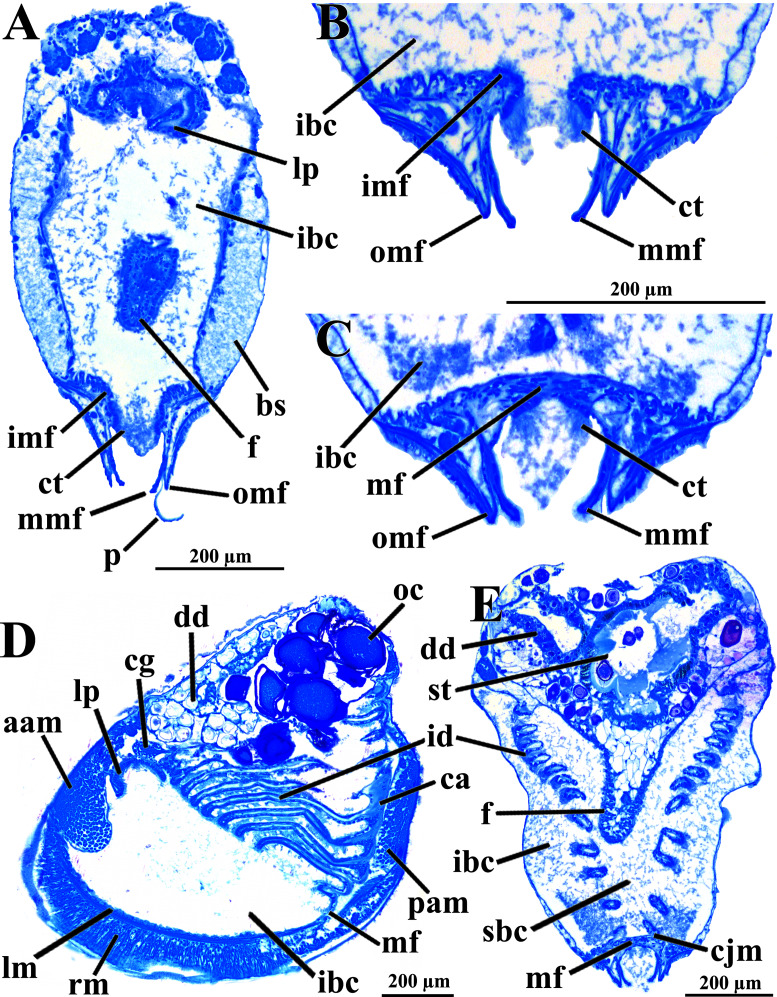
*Warrana besnardi—*Anatomy, as viewed by transversal (A, B, C, E) and parasagittal (D) histological sections. (A) Section through anterior part of infrabranchial chamber, showing blood spaces inside the mantle lobes, with their characteristic spongiform aspect. (B, C) Detail of the mantle margin lobes, showing free (B) and fused (C) inner folds. (D) Parasagittal section following the left mantle lobe margin, showing its radial and longitudinal musculature. (E) Section through median part of the animal at the level of fusion of the inner mantle margin; note the ctenidia fused to the mantle margin. See Fig. 1C for position of transversal planes. Abbreviations: aam, anterior adductor muscle; bs, blood space; ca, ctenidial axis; cg, cerebral ganglion; cjm, ctenidium joining the mantle margin; ct, ciliary tract; dd, digestive diverticulum; f, foot; id, inner demibranch; ibc, infrabranchial chamber; imf, inner mantle fold; lm, longitudinal muscles; lp, labial palp; mf, mantle fusion; mmf, middle mantle fold; oc, oocyte inside the ovary; omf, outer mantle fold; p, periostracum; pam, posterior adductor muscle; rm, radial muscles; sbc, suprabranchial cavity; st, stomach.

The mantle lobes are thin, except in their antero-ventral half extension, where the inner and outer epithelia are separated by a spongiform tissue, here interpreted as a large blood space (Fig. 3A). This tissue occupies a great area extending from the top of the infrabranchial cavity to the margin of the mantle lobes, tapering posteriorly and ending at the point where the mantle lobes are fused. The mantle margins lobes have the three typical folds of bivalves (external, median and internal) (Figs. 3A–3C). The median fold forms a narrow crest and has cilia that are probably sensory, while the inner fold is higher and appears strongly pleated caused by contraction in the preservative. A narrow continuous ciliary tract runs along the inner mantle fold, from the vicinity of the anterior adductor muscle to the posterior fusion of the mantle lobes. Fine radial muscular fibers extend to the mantle margins, penetrating the three folds (Fig. 3D). In addition to this musculature, there are also longitudinal muscle bundles running parallel to the mantle margin.

Ventrally, the margins of the mantle lobes are widely separate, except for one ventral point, where the inner marginal folds fuse with each other (fusion “type A” of Yonge, 1957, 1982) separating a narrow, postero-ventral exhalant aperture from the broader antero-ventral inhalant-pedal gape (Figs. 2 and 3C–3E). There are no papillae or tentacles in any of the three folds. There are no siphons and so the pallial line is entire.

**Figure 4:**
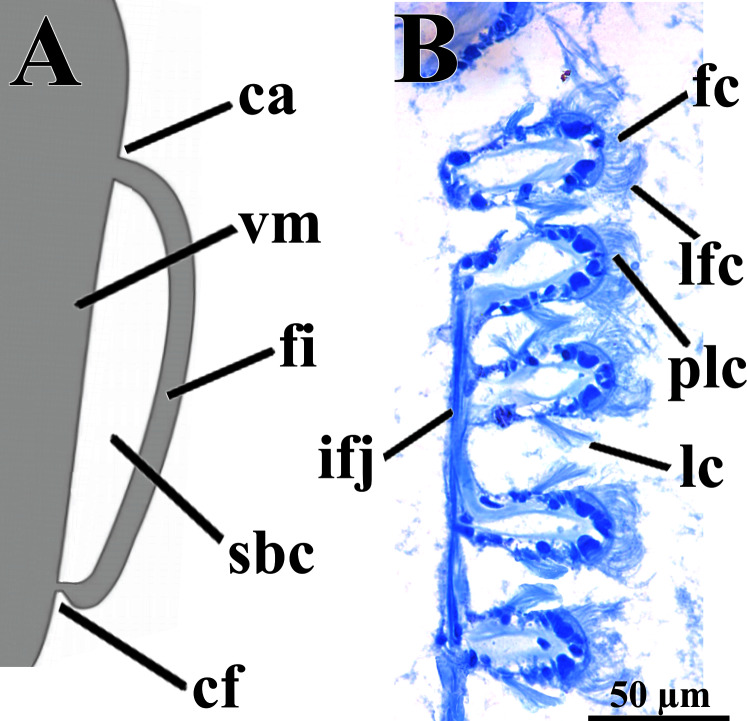
*Warrana besnardi*—Ctenidia. (A) Diagrammatic view of one filament, with its single descending part; as a more “dorsal” filament, its distal tip shown fused to the visceral mass epithelium. (B) Five filaments in transverse section. Abbreviations: ca, ctenidial axis; cf, ctenidial fusion to the visceral mass; fc, frontal cilia; fi, filament; ifj, interfilamentar junction; lc, lateral cilia; lfc, laterofrontal cilia; plc, prolatero-frontal cilia; sbc, suprabranchial cavity; vm, visceral mass.

The eulamellibranch, homorhabdic and smooth ctenidia of *W. besnardi* are incomplete and strongly reduced, each one formed by a single (the inner) demibranch, which is composed of only the descending lamella (Figs. 2, 3D, 3E, and 4A). The ctenidial axis is almost vertically positioned and does not reach the umbonal cavity. Up to twelve branchial filaments extend anteriorly, bearing frontal, prolatero-frontal, latero-frontal, and lateral cilia, and are internally reinforced by a fusiform chitinous rod (Fig. 4B). The filaments are joined by about three or four rows of very fine interfilamentar junctions, and the distal tips of adjacent filaments are fused; a marginal food groove is absent. The most dorsal filaments have their distal tips connected to the visceral mass by tissue junction (Fig. 4A), but all other filaments are free, except the most ventral ones, which are fused to the tissue formed by the fusion of the mantle margins (Figs. 3D–3E). Without interlamellar junctions, the cavity between the demibranchs and the visceral mass epithelium forms the suprabranchial chamber, which is not ample due to the low number of filaments; it communicates with the wide infrabranchial chamber. The most dorsal filament is the longest, borders the distal oral groove and is inserted between the labial palps, which constitutes an association of type II of Stasek (1963). Strongly contracted in the chemically fixed examined animals, the labial palps are barely visible and appear to be very small (Fig. 2).

**Figure 5:**
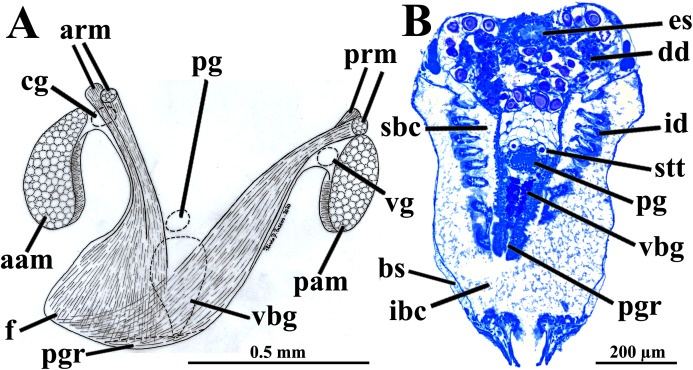
*Warrana besnardi*—Foot. (A) Diagrammatic view from the left side, showing the ganglia, reduced byssus gland, and pedal ventral groove. (B) Transverse histological section showing statocysts (see Fig. 1C for position of section plane). Abbreviations: aam, anterior adductor muscle; arm, anterior retractor muscles; bs, blood space; cg, cerebral ganglion; dd, digestive diverticulum; es, esophagus; f, foot; id, inner demibranch; ibc, infrabranchial cavity; pam, posterior adductor muscle; pg, pedal ganglion; pgr, pedal groove; prm, posterior retractor muscles; sbc, suprabranchial cavity; stt, statocyst; vbg, vestigial byssal gland; vg, visceral ganglion.

**Figure 6:**
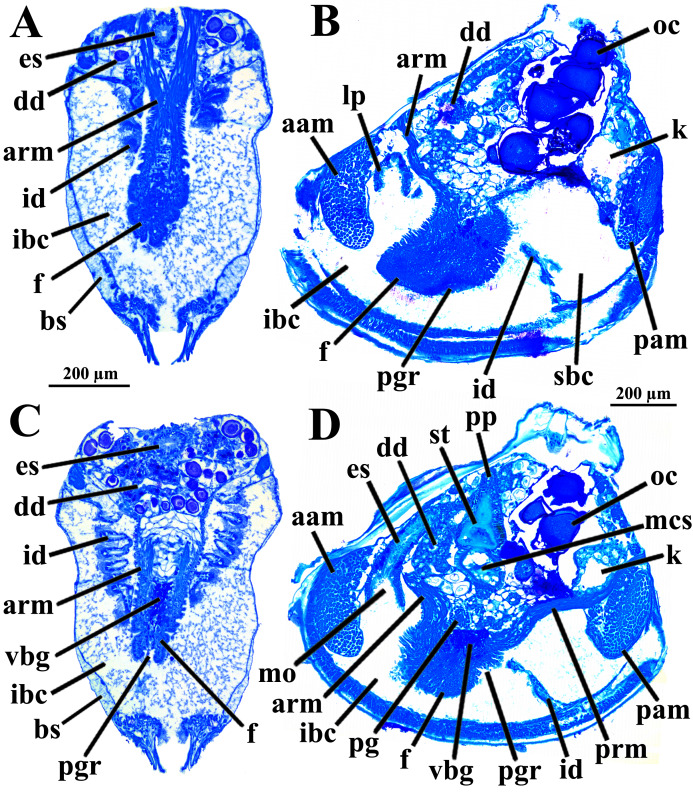
*Warrana besnardi*—Transversal (A, C) and parasagittal (B, D) histological sections showing foot (including vestigial byssal gland and associated pedal groove, pedal ganglion, and musculature), digestive system, and kidney. See Fig. 1C for position of transversal planes. Abbreviations: aam, anterior adductor muscle; arm, anterior retractor muscle; bs, blood space; dd, digestive diverticulum; es, esophagus; f, foot; id, inner demibranch; ibc, infrabranchial cavity; k, kidney; lp, labial palp; mcs, combined midgut and crystalline style-sac; mo, mouth; oc, oocyte inside the ovary; pam, posterior adductor muscle; pg, pedal ganglion; pgr, pedal groove; pp, dorso-posterior projection of the stomach; prm, posterior retractor muscle; sbc, suprabranchial cavity; st, stomach; vbg, vestigial byssal gland.

The foot is relatively large, projected ventro-anteriorly; in its chemically fixed state, it was always contracted inside the pallial cavity (Fig. 1). It is pointed distally, bearing a weak proximal heel and no ventral sole (Figs. 1, 2 and 5A). In its distal half, the latero-ventral epithelium is densely ciliated, whereas in the dorsal and proximal epithelia cilia are absent. A reduced, non-functional byssal gland is present and opens into a short ventral pedal groove whose lateral walls also do not have cilia (Figs. 5B, 6B–6D); byssus threads were not observed. Dorso-laterally to the pedal ganglia lie a pair of statocysts, each one being a capsule lined by cuboid cells and bearing a single, large (about 12 µm in diameter), and rounded statolith (Fig. 5B). The presence of hair cells in the epithelium of the capsule could not be confirmed. These statocysts can be classified as the “Type B1” of Morton (1985).

There are two pairs of well-developed retractors, one anterior and another posterior (Figs. 5A and 6); protractor muscles are absent. The fibers of the anterior retractors are attached to the valves close to but separated from the placement of attachment of the anterior adductor muscle, and from this point they extend ventrally as separate sheaths (Fig. 5A), with some fibers from the left bundle passing to the right, and vice versa (Figs. 6A–6C), and eventually forming the inner and distal pedal musculature. The posterior retractor muscles are attached to the valves dorsally and contiguous to the posterior adductor muscle (Figs. 5A and 6D). From this point of insertion, they promptly fuse as a single bundle that penetrates the foot, forming an external layer of musculature.

**Figure 7:**
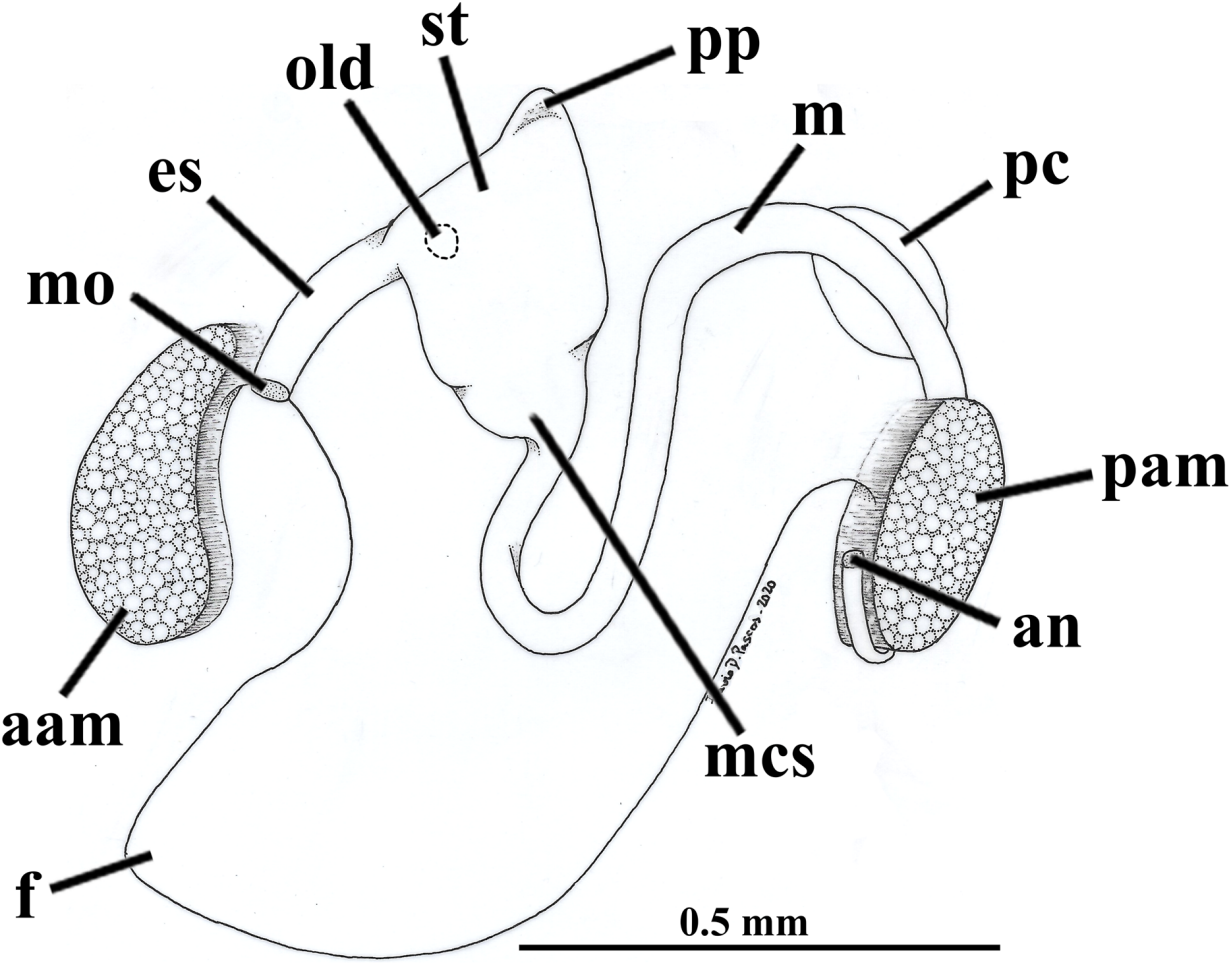
*Warrana besnardi*—Digestive tract, as viewed from the left side. Abbreviations: aam, anterior adductor muscle; an, anus; es, esophagus; f, foot; m, midgut; mcs, combined midgut and crystalline style-sac; mo, mouth; old, opening of the left digestive diverticulum; pam, posterior adductor muscle; pc, pericardial cavity; pp, dorso-posterior projection of the stomach; st, stomach.

**Figure 8:**
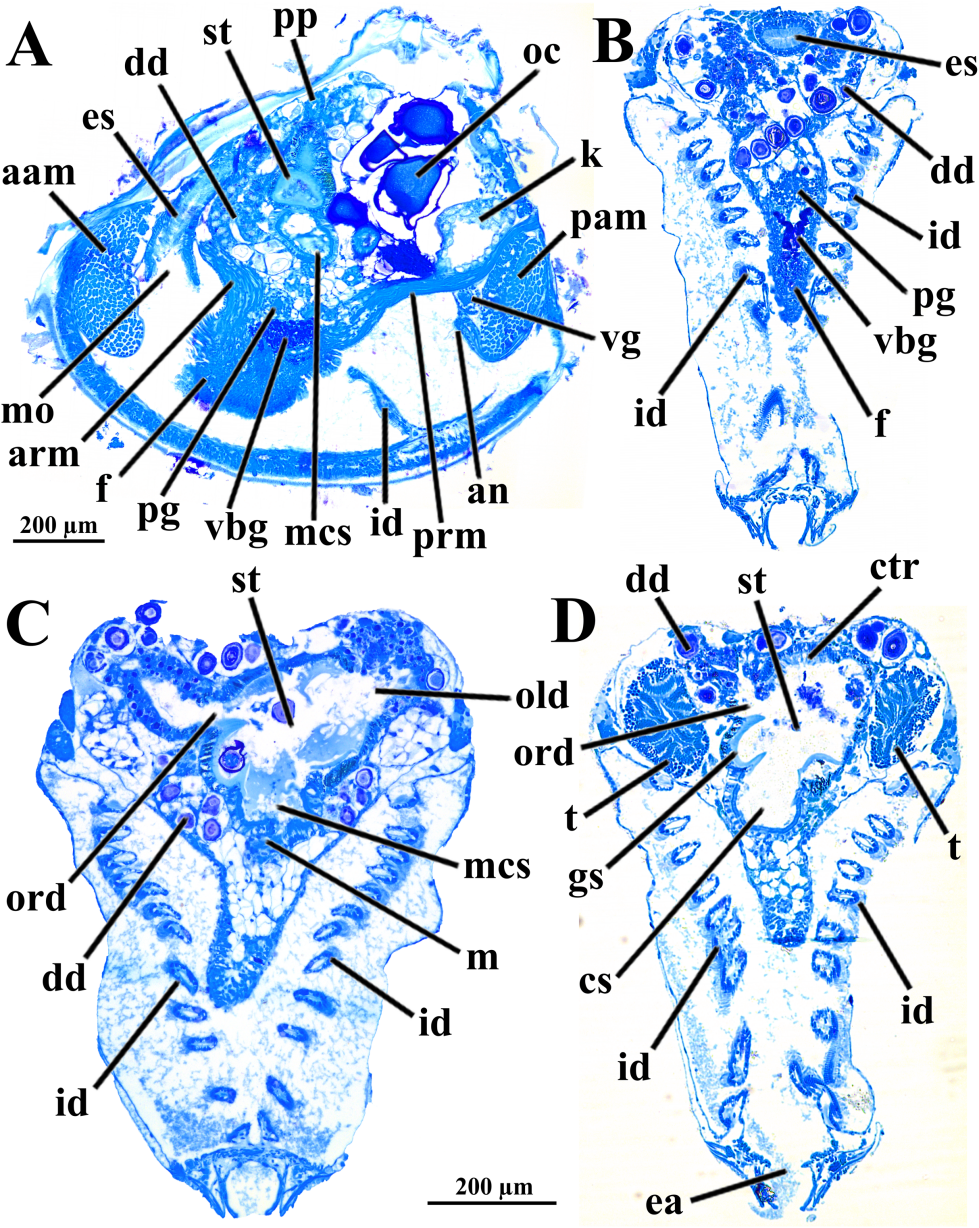
*Warrana besnardi*—Sagittal (A) and transverse (B–D) histological sections, showing the digestive tract. The transversal plane of C is indicated in Figs. 1C; 1B and 1D were obtained from a male, and the approximate position of their planes are also indicated in Fig. 1C; the transverse sections are all at same scale. Abbreviations: aam, anterior adductor muscle; an, anus; arm, anterior retractor muscle; cs, crystalline style-sac; ctr, ciliary tract of the stomach roof; dd, digestive diverticulum; ea, exhalant aperture; es, esophagus; f, foot; gs, gastric shield; id, inner demibranch; k, kidney; m, midgut; mcs, combined midgut and crystalline style-sac; mo, mouth; oc, oocyte inside the ovary; old, ord, opening of the left and right digestive diverticula, respectively; pam, posterior adductor muscle; pg, pedal ganglion; pp, dorso-posterior projection of the stomach; prm, posterior retractor muscle; st, stomach; t, testis; vbg, vestigial byssal gland; vg, visceral ganglion.

The mouth is relatively large, flanked by the reduced labial palps (Figs. 2, 3D, 6B and 6D). It is continuous with a relatively short and wide esophagus that opens into the dorso-anterior wall of the stomach (Figs. 6D and 7) and bears a ciliated, smooth inner epithelium (Figs. 8A and 8B). The stomach is a relatively capacious organ (Figs. 3E, 6D and 7) and each antero-lateral wall communicates with a large duct that branches and originates the tubules of digestive diverticula (Figs. 8C and 8D), which are bulky and occupy the antero-dorsal part of the visceral mass (Figs. 3D, 5B, 6B–6D, 8A, and 8B). Internally, the stomach has a well-developed gastric shield, and a densely ciliated epithelium in the roof (Fig. 8D). No areas of selection or typhlosoles were observed. Dorso-posteriorly, there is a projection of the stomach wall (Figs. 6D, 7, and 8A). The combined midgut and crystalline style-sac leave the stomach mid-ventrally (Figs. 6D, 7, and 8C). The style sac is relatively wide and short (Fig. 8D), and no crystalline style was observed in any sectioned individual. The midgut extends ventrally into a broad coil before turning backwards and upwards; it then turns downwards passing through the thin walls of the pericardial cavity and ventricle that are positioned posteriorly in the visceral mass (Fig. 7). The rectum passes close to the posterior and ventral side of the posterior adductor muscle ending in the anus (Figs. 7 and 8A).

**Figure 9:**
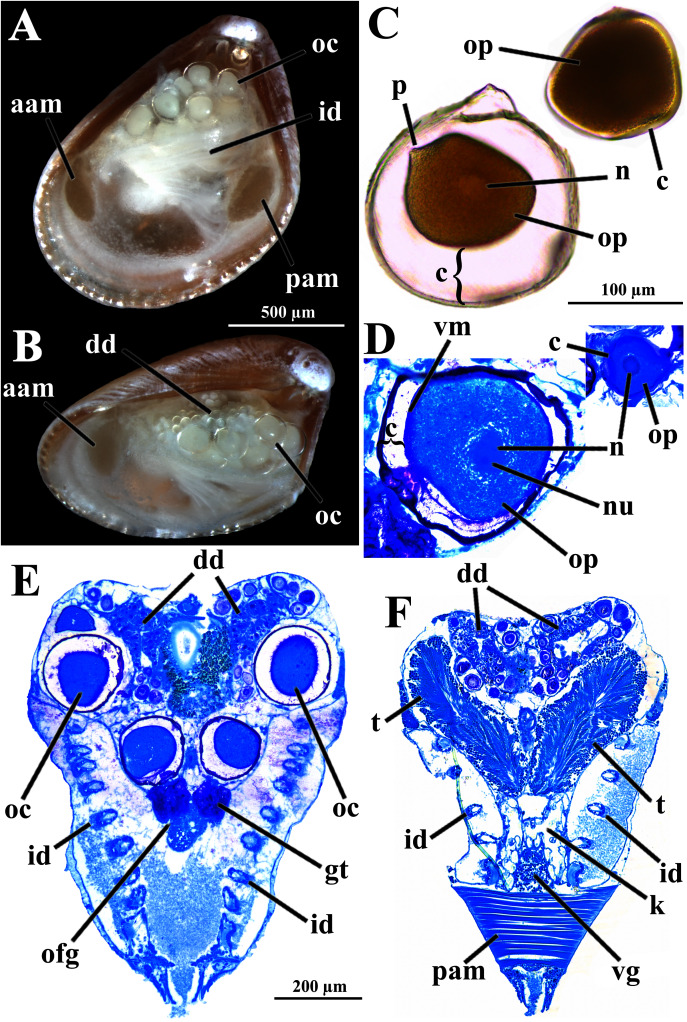
*Warrana besnardi*—Reproductive characteristics. (A, B) Oocytes *in situ* in a female with left shell valve removed. (C, D) Details of oocytes, with their characteristic coating; oocytes in C extracted from dissected individual, in D from histological sections. (E, F) Transverse histological sections of a female and a male, respectively (see Fig. 1C for position of transversal planes). A and B, C and D, and E and F at same scale. Abbreviations: aam, anterior adductor muscle; c, capsule; dd, digestive diverticulum; id, inner demibranch; gt, glandular tissue surrounding the opening of the female gonad; k, kidney; n, nucleus; nu, nucleolus; oc, oocyte inside the ovary; ofg, opening of the female gonad; op, ooplasm filled with yolk granules; p, peduncle; pam, posterior adductor muscle; t, testis; vg, visceral ganglion; vm, vitelline membrane.

The histologically studied specimens showed separate sexes (Figs. 1 and 9), and so *W. besnardi* is a gonochoric or a consecutive hermaphroditic species. There is one pair of gonads that in both males and females fills much of the posterior part of the visceral mass (Figs. 1A, 1B, 9E, and 9F). Dissected individuals larger than 0.8 mm in shell length had gametes in their visceral mass. When fully mature, the gonads spread anteriorly, lateral to the digestive diverticula (Figs. 8D and 9B). The ovary is a large and elongate follicle, producing large oocytes up to 120 μm in diameter (Figs. 9C and 9D); 23 were counted inside the ovaries of the female sectioned through the sagittal and parasagittal planes, and 15 in the other that was sectioned transversely. Each oocyte is richly furnished with yolk granules of about three μm in diameter that surround the central nucleus. A thin (four μm thick) vitelline membrane surrounds the oocyte. This in turn is covered by a capsule formed by a smooth hyaline coat of variable thickness (to 74 μm) that has a tough outer membrane. The oocyte has a short peduncle anchoring it to this outer tough membrane. The ovary releases the oocytes into the suprabranchial chamber by a pore placed just in front of the kidney; the gonopore is surrounded by a glandular tissue (Fig. 9E). The testes are large and elongate, bearing folded walls (Fig. 9F). The place where fertilization occurs could not be observed. None of the dissected or sectioned animals were found with broods.

The kidneys are saccular, partially fused to each other, and placed dorsal to the posterior adductor muscle (Figs. 6B, 6D, 8A and 9F). The nervous system has the three common paired ganglia found in other bivalves, and the three sets of ganglia are similarly sized. One cerebral ganglion is placed on each side of the mouth (Figs. 3D and 5A), and this pair is connected by a supraesophageal commissure. The pedal pair is partially fused and located at the base of the foot, behind the anterior pedal retractor muscles (Figs. 5, 6D, 8A and 8B). The visceral ganglia are partially fused and placed at the anterior side of the posterior adductor muscle (Figs. 5A, 8A and 9F).

## Discussion

**Figure 10:**
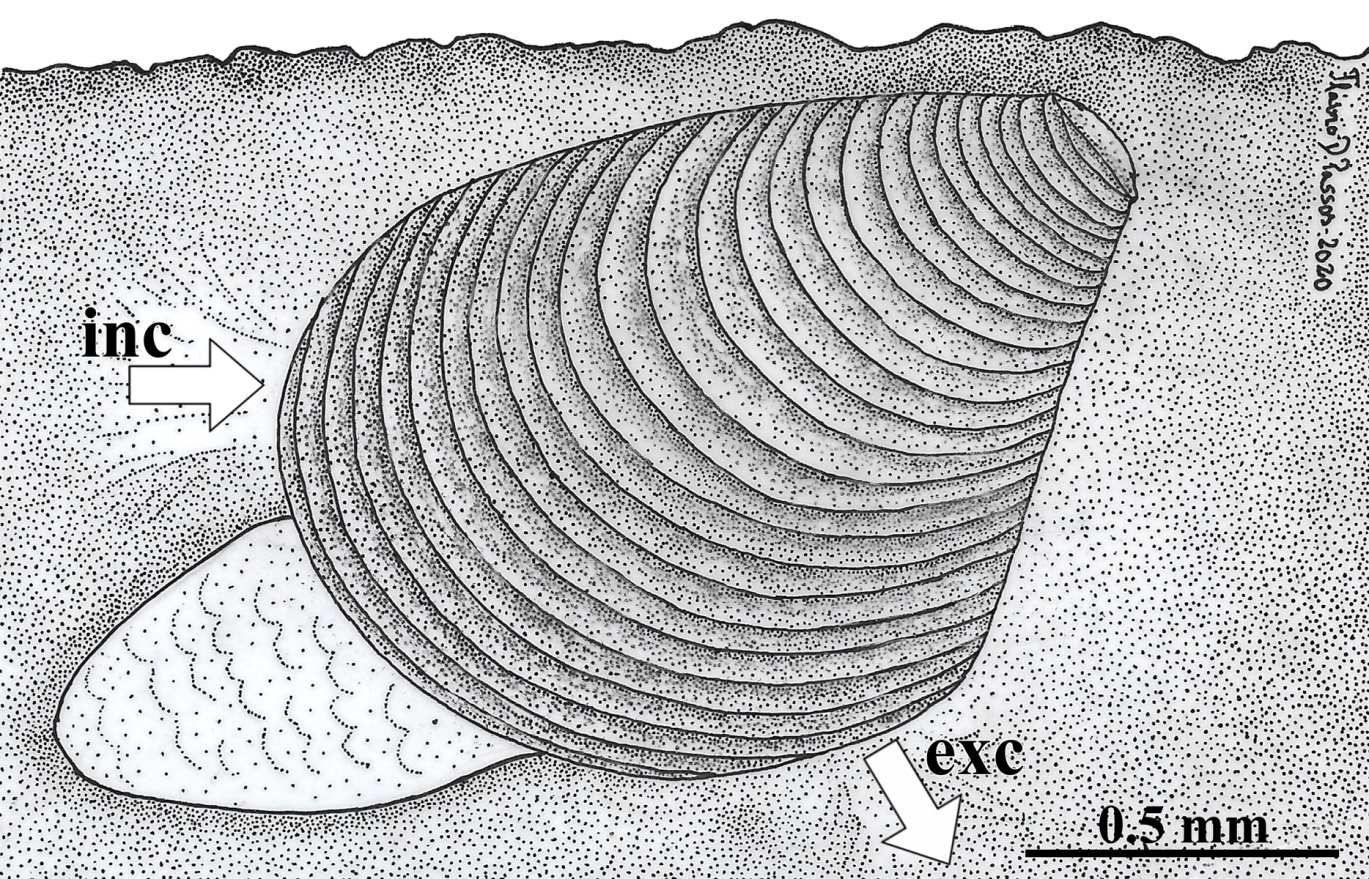
*Warrana besnardi*—Hypothesized position of the animal in the substratum. The presumed anterior and posterior water currents are indicated by arrows. As the examined specimens were not observed alive, the actual depth within the sediment remains unknown. Abbreviations: exc, exhalant current; inc, inhalant current.

Because the examined specimens were obtained from sediment samples, it is here concluded that *W. besnardi* is an infaunal bivalve species, as it was also recorded by Moore (1961) for *Goniocuna dalli*. The inferred position of the animals in the substrate is shown in Fig. 10. This figure also shows its hypothetical position as a burrower near the surface of the substratum, a behavior that is inferred from observations of the characteristics of its pallial complex and the absence of fouling organisms on its shell. Below, each of the main features of the mantle, ctenidia, foot, and of the organs of the digestive and reproductive systems are compared to the ones of other archiheterodonts, envisaging how this minute species lives, with special attention to its locomotion, feeding, and reproduction. Through this analysis, it will be discussed which of these features might relate to small size.

Among the Crassatelloidea, the Astartidae have been recorded as an infaunal group, living buried in muddy sand with gravel (Saleuddin, 1965), with shallowly burrowing forms at or near the surface having the posterior part of the shell settled by fouling organisms (*e.g*., as described by Salleudin for *Astarte sulcata* (da Costa, 1779)). Members of the Crassatellidae are epifaunal (Harry, 1966; Allen, 1968) or infaunal (Taylor, Glover & Williams, 2005). In the Carditoidea, Yonge (1969) described both infaunal (burrowing) and epifaunal (byssally attached) carditids, and González & Giribet (2012) recorded a semi-infaunal mode of life for a new carditid species and discussed the differences between this and epifaunal byssate species. All species of archiheterodonts examined by these prior authors were reported as suspensivorous, in which the food is brought by the inhalant water current. The infaunal and epifaunal species differ regarding the place where this current enters the mantle cavity: posteriorly in the former, anteriorly in the latter. In the four species of Astartidae studied by Saleuddin (1965), the water enters the mantle cavity below the point of mantle fusion that separates the exhalant aperture, and this inhalant area is separated from the pedal gape by only an apposition of the inner mantle folds. This condition is also found in the infaunal carditids and crassatellids, differing from the epifaunal byssally attached species of these families, in which there is an anterior inhalant current (Harry, 1966; Allen, 1968; Yonge, 1969; Taylor, Glover & Williams, 2005).

Like all other archiheterodonts, *W. besnardi* has two pallial apertures, but the position of the mantle margin fusion in this species is more ventral in this species than in other archiheterodonts, which is reflected in the expansion of the antero-ventral shell margin during growth (Batistão & Passos, 2020). As illustrated in Fig. 10, and because of the position of the mantle fusion, the inhalant water current must be anterior. An anterior inhalant current occurs generally in juvenile forms of Bivalvia (*e.g*., Ockelmann & Muus, 1978; Reid et al., 1992) and is often associated with small-bodied species (smaller than three mm; *e.g*., Passos, Domaneschi & Sartori, 2005; Passos & Domaneschi, 2006), and is here inferred as a paedomorphic feature for *Warrana* and may be related to miniaturization.

*Warrana besnardi* has a large antero-ventral area of its mantle lobes filled by a spongious tissue, interpreted here as a blood space. Taylor, Glover & Williams (2005) also recorded a similar blood sinus for *Eucrassatella donacina* (Lamarck, 1818), a large-bodied member of Crassatellidae (with shell lengths exceeding 120 mm), but in the latter species it is intimately associated with glands occurring near and parallel to the mantle margin lobes. As these mantle glands were widely reported for other Crassatellidae (Pelseneer, 1911; Harry, 1966; Allen, 1968), Astartidae (Saleuddin, 1965), and Carditidae (Yonge, 1969), they are here considered as lost in *W. besnardi* as a result of miniaturization in this species or its ancestor. In contrast, the blood sinuses may have been maintained as a compensation for the reduction of its gills, as discussed below.

When compared to other archiheterodonts, the ctenidia of *W. besnardi* are remarkably reduced in structure, with only one (the inner) demibranch on each side and each demibranch consisting of only the descending lamella; it bears few filaments; a food groove is absent. Complete ctenidia were described for larger-bodied species of Astartidae (*e.g*., Saleuddin, 1965, 1967), Crassatellidae (*e.g*., Harry, 1966; Slack-Smith, 1998; Taylor, Glover & Williams, 2005), and Carditidae (*e.g*., Yonge, 1969; Güller & Zelaya, 2013). In a small species of Astartidae (*Goodallia triangularis* (Montagu, 1803), with a maximum shell size of four mm), only a single demibranch (the inner) is present (Saleuddin, 1965); this condition also occurs in species of *Crassinella* (Crassatellidae) (Harry, 1966; Allen, 1968). For species classified as condylocardiids, reduction to a single demibranch was recorded by Middelfart (2002a, 2002b), Oliver & Holmes (2004) and Güller & Zelaya (2013) (in this latter work, a species of the *Carditopsis* was reported as having a single demibranch, whereas two species of *Carditella* were recorded as bearing both demibranchs, with the outer strongly reduced). Reduction of ctenidial elements such as demibranchs or filaments is a recurrent phenomenon in Bivalvia, occurring independently in many groups (*e.g*., Purchon, 1939; Bieler et al., 2014, MorphoBank matrix), and frequently associated with small size (as discussed for species classified as condylocardiids by Güller & Zelaya, 2013). In *W. besnardi*, associated with the anterior inhalant current, this reduction may have affected its feeding biology: we speculate that sediment particles enter the mantle cavity by this current and/or the animal’s burrowing activities and the ctenidia and as such associated reduced labial palps might be expected to play only a minor role in food capture and selection. This leads to the interpretation of this species as a passive deposit feeder—a novelty for archiheterodonts as all other studied species are suspension feeders – and this appears causally linked to miniaturization.

The foot is well developed in the species of Astartidae with infaunal habits (Saleuddin, 1965), as well as in the Carditidae of infaunal and epifaunal byssally attached habitat (Yonge, 1969). For smaller-bodied Crassatellidae (with adult sizes reaching 3.5 and 8 mm, respectively), Harry (1966) described the foot as long and digitiform, with a climbing ability and a functional byssus gland, and thus with an epifaunal mode of life, as was also reported by Allen (1968). For larger species of this family (exceeding 120 mm), Taylor, Glover & Williams (2005) described an infaunal burrowing habit. As the foot of *W. besnardi* was only observed in chemically fixed animals, its activity could not be verified, but its overall form, the small non-functional byssal gland, the well-developed retractor musculature, as well the structure of the statocysts are all characters suggestive of an actively burrowing bivalve. Facilitated by the strong commarginal sculpture of the shell, *W. besnardi* may dig next to the surface, with sediment particles entering the pallial cavity anteriorly while it moves forward by the combined ciliary and muscular action of the foot. Adult *W. besnardi* do not retain a functional byssus gland, as it has occurred in some other bivalve lineages.

When compared to other archiheterodonts, the digestive organs of *W. besnardi* exhibit some degree of simplification, here also considered largely influenced by miniaturization. The esophagus has a smooth inner epithelium, whereas it has longitudinal folds in species of *Astarte* (Saleuddin, 1965, 1967). In the stomach, no areas of selection or typhlosoles were observed, and there are only two large ducts communicating with the digestive diverticula, one on each side. In the stomach of Astartidae (Purchon, 1958; Saleuddin, 1965) and Carditidae (Purchon, 1958; Yonge, 1969), sorting areas are present, the typhlosoles are well developed, and the wall of the stomach is folded, bearing two or many ducts of the digestive diverticula opening into the lumen. In the Crassatellidae, Allen (1968) related small body size within the family to the absence of ciliated sorting areas, the small size of the dorsal hood and the small (two) number of apertures leading from the stomach to the digestive diverticula. The storage vesicles found among the organs of the visceral mass of the Astartidae (Saleuddin, 1965, 1967), but not in the Crassatellidae (Allen, 1968), were not observed in *W. besnardi*.

Regarding the reproductive traits of archiheterodonts, brooding protection is one of the most cited characteristics. This is a common feature for the Carditidae (*e.g*., Dall, 1902), and led Yonge (1969) to state that “in certain, possibly all, species (of Carditoidea) the young are incubated”. Ovoviviparity is also a prevalent character of carditids, with broods (reported as ranging from 14 to 93 by Jones, 1963) held among the gill filaments and released as juveniles (*e.g*., up to 1.29 mm in shell length *teste* Jones, 1963; or up to 0.8 mm *teste* Yonge, 1969). Moreover, maternal care is exhibited after brood protection, with young being found byssally attached to adults (Jones, 1963), and incubation culminating in extra-body brooding pouches formed by infolding of the valves, forming a “marsupium” (Dall, 1902; Yonge, 1969). Schneider (1993) suggested that brooding may be the primitive condition for the carditids. For the condylocardiids, observations are mainly restricted to the information provided by Bernard (1896a, 1896b), Moore (1961), Waller (1973), Salas & Rolán (1990), Salas & von Cosel (1991) and Middelfart (2002a, 2002b). Moore (1961) was the only author to record ovoviviparity in a living condylocardiid, and Salas & von Cosel (1991), Middelfart (2002a) and Oliver & Holmes (2004) observed chemically fixed animals with brooded juveniles, albeit in different places of the parent’s body: in two specimens of *Carditopsis gofasi* Salas & von Cosel, 1991, the young appeared to be held among the gill filaments (Salas & von Cosel, 1991; figs. 42–47). This condition was also illustrated for *Condylocardia notoaustralis* Cotton, 1930 by Middelfart (2002a: fig. 6), whereas a “a single larva, about 350 µm in diameter” (presumably almost a juvenile) was described as incubating inside the infrabranchial chamber of an individual of *Condylocuna io* (Bartsch, 1915) by Oliver & Holmes (2004: fig. 140). Based on the prodissoconch size, Batistão & Passos (2020) suggested that *W. besnardi* has lecithotrophic development and suggested that it broods its young. In the present study, as no embryos or juveniles were found, brooding protection was not observed.

Another striking feature present in archiheterodonts, characteristic of lecithotrophic and brooding species, is the occurrence of yolky oocytes. These oocytes are smaller in Astartidae and Crassatellidae. Size ranges include, *e.g*., 150–200 μm in diameter in the four larger and 110–120 μm in one smaller species examined by Saleuddin (1964), 200 μm in *Crassinella* spp. studied by Harry (1966), and up to 375 μm in Carditidae (Yonge, 1969). In these cases, each oocyte is surrounded by a coat, of variable width in the different families: from only 15 μm in Crassatellidae (Harry, 1966) and 20–25 μm in Astartidae (Saleuddin, 1964), to 90 μm in Carditidae (Yonge, 1969). The oocytes of *W. besnardi* are comparable to the former two families in size (about 120 μm in diameter), although its coat is wider (74 μm in width) than reported from Carditidae. In view of the strong reduction of the ctenidia of *W. besnardi*, its oocytes appear to be too large to be incubated in the small suprabranchial chamber. This extreme miniaturization could mean: (1) this species does not invest in protection of its broods inside the maternal body, and the (fertilized or unfertilized) oocytes are released directly to the environment to complete their development; or, (2) its oocytes are fertilized within the maternal body and brooding takes place for a short time, or up to the juvenile phase (ovoviviparity), but in this latter case with a small (ca. 20 or fewer) number of broods. The first hypothesis is supported by the investment in a thicker, durable coat, as it would function as a protective capsule against predators, mechanical impact, or attack of microorganisms, as has also been suggested for other bivalves (Chérel, Beninger & Le Pennec, 2020). The second hypothesis is supported by the presence of a communication between the supra- and infrabranchial chambers, providing a possibility for broods to pass through the ctenidia to be incubated in the infrabranchial chamber.

In the context of a comparative study on the anatomical features of the Carditoidea, Yonge (1969) discussed the diversification of the modes of life of its species, which, in his view, evolved initially from an ancestral form that had a globose, equivalve and equilateral shell. Yonge related this presumed primitive condition to an infaunal, burrowing, suspension feeding habit, still present in many recent species, and suggested that from this form the epifaunal, byssally attached lineages have arisen, including heteromyarian forms, albeit maintaining the original suspension feeding habit. At that time, relevant data on condylocardiids were not available.

As most, if not all, condylocardiid species have minute shell sizes, we conclude that miniaturization has strongly influenced the evolution and diversification of this group. A combination of plesiomorphic characters (single point of fusion of mantle margins, blood sinuses near mantle margins, a burrowing foot) and derived conditions potentially imposed by miniaturization (an inferred anterior inhalant current, reduced ctenidia, simplification of the digestive system parts, and possibly extra-body brood protection through coated oocytes) resulted in a new mode of life for Archiheterodonta: a passive deposit feeding habit. Additional studies on other species once classified as ‘Condylocardiidae’ will be necessary to learn whether this has occurred once or multiple times in the carditid **sensu* lato* clade.

## Conclusions

This is the first comprehensive anatomical study of a condylocardiid. The anatomical description of *Warrana besnardi* shows it to represent an extreme case of miniaturization. Many of the observed anatomical traits are interpretable as strongly influenced by the shift to a much-reduced adult size. Further comparative morpho-anatomical studies of miniaturized archiheterodont and other bivalves, as well increased resolution of lower taxonomic levels of the bivalve tree are needed to put the many recognized instances of bivalve miniaturization into a sound phylogenetic and macroevolutionary context.

## Additional Information and Declarations

### Data Availability

The following information was supplied regarding data availability: This is a descriptive study based on direct anatomical observations. The studied specimens are deposited in the Museum of Zoology of the University of Campinas (ZUEC): ZUEC-BIV 2623, 2625, 2626, 2629, 2632, 2639, 2646, 2648 to 2652, 2654, 2657, 2663, 2666, 2667, 2686, 2688, 2691, 2692, 2695, 2698 to 2700, 2702, 2703 and 5618, including the slides with the histological sections (ZUEC-BIV 8392 to 8396).
